# Entrepreneurship and Resilience in Spanish Sports Clubs: A Cluster Analysis

**DOI:** 10.3390/ijerph18105142

**Published:** 2021-05-12

**Authors:** Paloma Escamilla-Fajardo, David Parra-Camacho, Juan Manuel Núñez-Pomar

**Affiliations:** Department of Physical Education and Sports, Faculty of Physical Activity and Sport Sciences, University of Valencia, 46010 Valencia, Spain; paloma.escamilla@uv.es (P.E.-F.); david.parra-camacho@uv.es (D.P.-C.)

**Keywords:** entrepreneurial orientation, sports clubs, business model adaptation, COVID-19, sport management, performance

## Abstract

Entrepreneurial orientation can be an effective response by sports clubs to manage a recession, such as the COVID-19 crisis. Therefore, its study can be fundamental to understand different ways of managing a recession. This study analyzes the entrepreneurial orientation of Spanish non-profit sports clubs to identify different groups and their profiles. The sample is composed of 145 Spanish non-profit sports clubs. Different validated scales have been used to analyze entrepreneurial orientation, business model adaptation, service quality, and economic and social performance (performance in social impact and performance in social causes). Entrepreneurial orientation is the variable used to differentiate the groups. This is made up of three dimensions: innovation, risk-taking, and proactivity. According to the results obtained, there are three groups of sports clubs according to their entrepreneurial orientation: non-entrepreneurs (*n* = 11), moderate entrepreneurs (*n* = 85), and strong entrepreneurs (*n* = 45). There are substantial differences between the three groups according to the adaptation of the business model, the perceived impact of COVID-19, and the returns analyzed. Strong entrepreneurs have considerably higher levels of business model adaptation, economic performance, social performance, and perceived service quality than non-entrepreneurs. Theoretical and practical implications have been drawn that can bring new information to the sports and organizational sector. For example, the diagnosis of the different profiles according to the level of entrepreneurship can be useful to propose strategies to improve performance. In this way, it can help to evaluate the return on the investment made by sponsors or governments in the organization.

## 1. Introduction

Non-profit sports clubs have several social functions from a health perspective. Firstly, one of the responsibilities of these types of entities is to facilitate access to sports practice for all types of groups [1], so that they can improve their health and well-being at all levels. On the other hand, sports clubs have a fundamental role in the promotion and encouragement of physical activity and sport within society [2]. Therefore, these types of organizations are health promoters at two different and complementary levels. On the one hand, they develop health promotion at the individual level during the development and continued practice of physical activity and sport [3]. However, they go a step further by generating changes in communities related to their active behavior that have an important impact on the health and well-being of the practicing population [4].

However, the situation of COVID-19 has greatly altered the way people act and the way sports organizations operate [5]. The social restrictions and limitations of approach that still exist in most countries have forced people to change their sporting habits and their health and well-being being affected. In Spain, non-professional sports activity has been greatly affected during the last year, without having yet recovered the values reached before the virus. Numerous measures have been taken by governments and institutions with the main objective of curbing the infection curve [6].

All these limitations and restrictions have had a high impact on today’s society and sport has not been unaffected [5]. During this period of confinement, physical activity has been analyzed, concluding that the numerous restrictions and limitations have considerably reduced sports practice [7]. As expected, their limitation can have as a consequence a detriment in the quality of life of people since physical activity has a known positive effect on our health [8]. In addition, sports clubs have had to divide their efforts between maintaining health promotion and achieving economic sustainability to ensure the organization’s survival.

The limitations and important restrictions have also had an impact, as is logical, on sports organizations, which continue to face a major challenge. Sporting events and competitions have been considered important sources of virus infection [9]. This was one of the reasons why, during the first months of the pandemic, sporting events and leagues were cancelled, physical activity outside the home was limited, and the sports sector in general suffered a major impact [10].

Sports organizations, highly affected by restrictions and limitations, are still struggling today to meet the expectations and needs of their members and users, so that they are able to maintain their sustainability and guarantee their survival [11]. To this end, regardless of their nature, they have been forced to adapt and reinvent themselves in a way that is consistent with the current situation and with their main social objectives. The health promotion through the practice of sports and the facilitation of access to sports for all groups make more sense than ever in this situation [7]. For this reason, sports clubs have been forced to a large extent to reinvent themselves and adapt their business model to continue offering sports that improve the quality of life of their users.

If the focus is on non-profit sports organizations, in Spain, and according to the latest official data published, in 2019 there were a total of 75,455 federated sports clubs and a total of 3,945,510 federative licenses [12]. Generally speaking, this type of sports organization represents a very high percentage of the total number of sports organizations operating in any community. Thus, if Spain has in 2021 a total population of 47,351,567 inhabitants [13], and the latest report on sports habits states that 53.5% of the population practices physical activity either periodically or occasionally [14], 15.57% of that active population does so in sports clubs. Due to this high representation in the sports offer, which includes a large number of people, it is important to study and develop this type of sports organizations at an economic, social and sports level. The physical and sporting activity of a large number of people depends to a large extent on its sustainability and survival. The prominent role of this type of organization makes it especially important to analyze how all these recent limitations have affected their activity. Many members and athletes have had to completely change their sporting habits and customs, physical activity in person, organized and collective training, and sporting competition. However, can sports organizations do anything to maintain sports practice levels?

Sports clubs have been forced to evaluate new alternatives and innovate in order to be able to continue to offer sporting activities [11]. The importance of these initiatives, and especially their success, is due to the high impact that the work of these types of organizations has on society [15]. This social impact can be maintained if it is possible to give continuity to physical activity and sports training programs and, thus, to the quality of life and health of users and athletes. Moreover, they are not only concerned about their economic sustainability, but also about maintaining their social value in a difficult situation for the physical and mental health of the population.

However, crisis contexts require a response from the organizations [16]. Entrepreneurship plays an important role in this organizational response, offering different alternatives that, if managed effectively, can help to overcome this crisis. What is undeniable, though, is that this virus has largely modified the way organizations operate, and innovations and adaptations have been—and continue to be—if not mandatory, then highly necessary to maintain the survival of the organization. These types of initiatives and changes have not been developed and implemented equally in all Spanish sports clubs [8]. These decisions may have been influenced by previous experience, the level of impact perceived by COVID-19, the possibilities or resources available to the organization, expectations or needs of members and users or the course of action to be taken in the face of this crisis. In general, in stages of crisis or recession, entrepreneurial orientation can be considered as a management tool to handle them. Due to this, the present study focuses on analyzing entrepreneurial orientation by generating different profiles. This can provide useful and novel information to the sports field and the organization sector.

## 2. Theoretical Framework

### 2.1. Entrepreneurial Orientation

In this context of change and uncertainty, entrepreneurial and change management variables have been considered by sports organizations. Within this field, entrepreneurial orientation (EO) has been widely used as an important organizational attribute that includes “entrepreneurial top management style, organizational configuration, and new entry initiatives” [11] (p. 640). EO as a top management style focuses on the beliefs, objectives, decisions and the communication that top management demonstrates with the organization. On the other hand, EO as an organizational configuration is oriented towards creating organizational processes that facilitate a pattern of entrepreneurial behavior. Finally, EO as a new entry initiative is based on behavior oriented to the exploration and exploitation of external opportunities to create new value in the market [17].

However, EO does not generate an absolute consensus on its definition, nor on its constituent dimensions. There are two schools of thought based on EO: (i) The one that points out three dimensions, and (ii) the one that points out five dimensions [11]. The present study is based on Miller [18] and Covin and Slevin [19] who advocated three main dimensions: (i) Innovation, (ii) risk-taking, and (iii) proactivity.

Innovation is a constituent dimension of EO considered important in the growth and success of the organization [20]. This has been—and continues to be—the most analyzed and studied dimension of those that make up EO [21]. Innovation involves the transformation of knowledge and capabilities into new products or services, as well as the implementation of new transformation or production techniques [22]. This does not mean that they must be totally new initiatives for the sector, but that they must provide added value to the organization itself. There can be different types of innovations depending on whether they are based on existing skills or on the direction of the result. Product innovations are related to the modifications or initiatives developed in products or services according to the needs or expectations of users, and process innovations when changes are developed in the process of elaboration, proposal and delivery of value [23]. They may be related to innovations in the organization’s external relations, the production system, etc. Organizations often focus on identifying, assimilating and managing internal knowledge; however, it is also necessary to absorb and canalize knowledge that originates externally [24,25].

Risk-taking is the level of risk that the management of an organization assumes. For this, there must be a commitment of resources with the final idea that the benefit reported may be significantly higher than it would be if this risk were not assumed [26]. There are benefits that would never be achieved with a passive organizational behavior, however, it is necessary to know the balance between risk and benefit to avoid failure. The organizations are generally a reflection of their top managers [17]. The perception of risk is probably different for each manager according to his or her abilities, skills and previous experiences.

Proactivity is characterized by the exploration and exploitation of opportunities resulting in the development of initiatives ahead of the competition [27]. In order to carry out initiatives ahead of the competition, it is necessary to have a detailed knowledge of three important factors: (i) The tangible and intangible resources available to the organization itself and the possibilities in the short and medium term; (ii) the initiatives, actions and market trends being adopted by the direct competition; and (iii) the needs and expectations of current members and users and those of the potential market. In addition to the above, the speed and fit of the response can have a direct positive impact on the success of the organization. According to Laitinen [28], in situations of economic recession, the organizations most likely to succeed are those that act proactively, quickly, and adjust the offer to the members or user.

According to previous literature, in times of recession an entrepreneurial orientation can have a positive effect on macroeconomic variables such as development and employment [29]. In fact, it is important to generate an organizational change that generates agile environments that facilitate effective, constant, and proactive innovation [30]. Similarly, an entrepreneurial organization can generally have a better final performance than a conservative organization that does not implement innovations or initiatives adapting to the situation [31]. Hence, it is necessary to study and develop it in a hostile and dynamic situation such as the one that occurred after the irruption of COVID-19. Specifically, it may be interesting to know the entrepreneurial profiles of organizations, since it is a capacity or quality that may vary depending on the organization [32]. Depending on the profile and the entrepreneurial level, its subsequent performance may vary considerably. Therefore, it is necessary to study entrepreneurial profiles in periods of recession in order to draw broader implications that can improve the academic literature [33].

Finally, it is worth noting that the behavior of managers in charge of the organizations in the situation generated by the COVID-19 crisis can make a difference with respect to competition. Moreover, the way in which the recession is faced and the very survival of the organizations may depend to a large extent on the decisions made by the decision-makers [34]. Hence, it is important to analyze their perception of the environment and the decisions they make on this basis. The perception and identification of the impact that the COVID has had—and continues to have—on the organization is an individual aspect that can greatly affect subsequent actions. It is, therefore, important to consider it as a variable to be highlighted.

### 2.2. Business Model Adaptation

A final important dimension of analysis in a context of crisis such as the current one and complementary to the entrepreneurial orientation could be the adaptability of the business model. Entrepreneurial behavior and the adaptability of the business model may be related [34]. Business model adaptation (BMA) is the adjustment of the organization’s logic in the way it operates and creates value [35], and should be aimed at satisfying the unmet needs of current members and/or attracting new customers [36]: “In dynamic and changing environments such as the sports sector, it is necessary to adapt the business model according to the needs of users and partners, competition, technological changes, and social restrictions that limit communication channels” [37] (p. 3). BMA can be an effective response to situations that may have a high impact on the organization [38]. This adaptation can encompass actions and initiatives aimed at transforming an organization’s system of activity so that it is more in line with the environment [39].

Adapting the business model to the needs of the situation can ensure the survival of the organization [35]. However, there is no consensus in the academic literature on the dimensions that form this construct. Following Saebi et al. [40], it can encompass modifications in proposition, delivery and/or value capture. Thus, it would be important to identify the environment and adapt initiatives and actions to the current situation. In addition, knowing the needs and expectations of your target market can be vital for actions to be aligned consistently with what is expected or needed.

In times of crisis, would it be desirable for entrepreneurial organizations to adapt their business model? This research question has been previously addressed by the organizational literature [41]. In addition, does the adaptability of the business model provide relevant information on the organization’s ability to cope with the crisis? However, there is still little related body of knowledge in the field of sports.

### 2.3. Performance Variables

Non-profit sports clubs focus their activities and efforts to a greater extent based on their social responsibility, ahead of economic or financial aspects [26]. The social responsibility of this type of organization in Spain is based on encouraging, promoting and facilitating access to sports practice for all groups in society. Moreover, in this period of restrictions and limitations, sport has become a means and an end to improve the quality of life and health. Therefore, sports clubs have a social responsibility to strive to maintain a sports offer that can meet the needs and expectations of their members or users.

However, this social performance can be approached in two different but complementary ways [18]. On the one hand, social impact performance (SIP) “is aligned with a basic, and generally common, objective of sports clubs to achieve the greatest possible impact on the environment in terms of the number of members, programs developed, or knowledge and awareness in the community” [18] (p. 4).

On the other hand, social causes performance (SCP) is the social response by the sports club to facilitate the inclusion of minority groups or those at risk of exclusion. However, although financial objectives are not the most important goal for sports clubs, it is the economic aspect that guarantees, to a large extent, their sustainability and survival. Hence, it is necessary to achieve a balance between the creation of social value and economic value [42], and its analysis is fundamental. In this sense, despite its undeniable importance, the study of the economic aspect in non-profit organizations has generated controversy throughout the organizational literature [43].

Likewise, as far as is known, there are no previous studies that analyze different groups of entrepreneurs within sports organizations and their relationship with subsequent performance. Hence, the main objective of the study is to fill some of the gaps in the literature through two research questions. First, what groups exist in Spanish sports clubs considering their EO and what are the main characteristics that define their profile? Likewise, the second research question is based on knowing the degree of adaptation of the business model and the subsequent performance of each of the groups obtained.

## 3. Materials and Methods

### 3.1. Participants

The sample that participated in the study consisted of 145 Spanish sports clubs. Table 1 shows the organizational characteristics of the club and the individual characteristics of the managers analyzed. 55.17% (*n* = 80) are sports clubs with an international-national competition level, and considering the type of funding, 72.41% (*n* = 105) were mostly privately funded. On the other hand, considering the individual characteristics of the organization’s managers, 83.14% (*n* = 122) were men and 71.03% (*n* = 103) have higher or university studies. According to the position held in the organization, 74.49% (*n* = 108) were managers (president or board of directors). In terms of length of service in the sports club itself, 49% (*n* = 71) had been with the club for less than 10 years, while 48.28% (*n* = 70) had been working in sports sector for more than 21 years.

### 3.2. Instruments

The scale used to divide the participants into clusters was the entrepreneurial orientation scale created and validated by Covin and Slevin [19] and previously used in the sports context, with good psychometric data [9,22,44,45]. The scale consists of eight items divided into three dimensions. In the present study all dimensions also present good psychometric properties: (i) innovativeness (α = 0.71); (ii) risk-taking (α = 0.75); and (iii) proactiveness (α = 0.76). The overall reliability of the scale was 0.83.

To measure business model adaptation, there is no consensus in the literature. However, four aspects have been evaluated following Saebi et al. [40]. First, change in value proposition has been measured through two items. These have been analyzed separately in the present study since they consider opposite and very important aspects in the current context. Secondly, the change in target market was evaluated to determine whether the value proposition had been opened up to new customers and users. Thirdly, the change in value delivery was measured, focusing attention on whether the relationship with collaborators, suppliers or the reorganization of the club’s structure had been adapted. Finally, the change in value capture mechanisms was evaluated by considering changes in the price of products or services.

A scale created and validated by the authors [31] was used to measure economic performance. It is composed of four items that in the present study present good psychometric properties (α = 0.75). The scale to measure the service quality has been adapted from the scale previously created and validated in literature [46] and used in the sports context presenting good psychometric properties [11], in the same way as in the present study (α = 0.83). Similarly, to measure performance in social impact and performance in social causes, scales previously created and validated in the associative sports context were used with three and two items, respectively [26]. As in previous studies, they have good psychometric properties.

Finally, to determine the perceived impact of COVID-19 on the organization, the following question was asked: “To what extent has your club been affected economically or financially by the COVID-19 crisis?” in line with what has been proposed by specific literature based on times of crisis [40]. The response scale for the above variables was a Likert-type scale from 1 to 7, with 1 being “strongly disagree” and 7 “strongly agree”. However, in business model adaptation there was the possibility of answering 0 as “not undertaken”.

### 3.3. Procedure

Participants were contacted by telephone. In this first interview, the study was explained and collaboration was requested. Once the sports club accepted the collaboration, a more detailed letter of presentation of the study was sent via email which included a link to the online questionnaire hosted on the University of Valencia’s own platform (LimeSurvey). The main requirement was that the questionnaire be completed by a manager or person responsible for the organization with a global vision of the sports club over the last three years. The cover letter specified that the questionnaire would be anonymous and that the data would be processed for academic purposes only. The estimated response time was 15 min and the sample was collected between April and May 2020. The present study followed the guidelines of the Declaration of Helsinki. On the part of the academic institution, the Ethics Committee was approved with resolution code 1119833 by the University of Valencia (Spain).

### 3.4. Data Analysis

Statistical analyses were performed with the SPSS version 24.0 for Windows (IBM, Armonk, NY, USA). First, a cluster analysis was carried out including the eight indicators of the entrepreneurial orientation scale. Cluster analysis allows grouping individuals into clusters, allowing individuals in the same cluster to be more similar to each other than to individuals in other clusters, trying to maximize the homogeneity of the subjects within the cluster while maximizing the heterogeneity of the aggregates [47]. To obtain the cluster solution, the hierarchical estimation method was combined with the non-hierarchical K-means estimation method, a procedure recommended by Hair et al. [47] to optimize the solution. The hierarchical cluster analysis was performed using the Ward’s Method clustering process, using as similarity measure the Euclidean distance to the quantile to obtain the agglomeration history. The cluster solution obtained from the previous analysis was used to perform a non-hierarchical K-means analysis using as initial centers the means of the variables obtained for each cluster solution of the hierarchical analysis.

On the other hand, a discriminant analysis was performed to check the level of accuracy and validity of the proposed cluster analysis. In this analysis the dependent variable corresponds to the proposed cluster solution and the independent variables were the items of the entrepreneurial orientation scale. The data provided by SPSS for the Wilk’s lambda statistic, the canonical correlations, the chi-square value and the probability associated with each function were observed. The au-values provided by each function and the proportion of the variance explained by the correlation between the respective canonical variables were also observed. Finally, the classification matrix was observed to check the percentage of correctly classified cases.

To define the characteristics of the group profiles and evaluate the predictive validity, ANOVAS was performed, with application of the Bonferroni and Tamhane post-hoc test, in the absence of homogeneity of variances, and chi-square tests with the sociodemographic and variables of interest that were not included in the cluster analysis. The value of the contingency coefficient (C) was also used to test the strength of the association or the effect size of the related variables.

### 3.5. Assessing Common Method Bias

Both procedural and statistical methods were used to address the common method bias issue [48,49]. For example, anonymity and confidentiality of the respondents were ensured, and the survey questionnaire was pretested before the main data collection to ensure that there were no difficult or confusing items.

Additionally, a clear set of instructions was provided to facilitate easy completion of the survey. Additionally, this study adopted Harman’s [50] one-factor test to examine common method bias. All items were entered into a principal component analysis with Varimax rotation to determine whether a general factor or single factor accounted for more than 50% of the covariance. The results of analysis indicate that the first factor explain only 46% (below of 50%) of the variance, suggesting that common method bias has no impact on the present study [51].

## 4. Results

### 4.1. Identification and Description of Clusters

Hierarchical analysis was used to observe the increases in the agglomeration coefficient among possible cluster solutions and to obtain the initial cluster centers. After analyzing the increases in the agglomeration coefficient, it was decided to test the two-, three-, four-, and five-cluster solutions with the application of the non-hierarchical K-means method, using the initial centers of the hierarchical analysis. It was decided to contrast all possible solutions due to the absence of previous studies on entrepreneurial orientation in sports clubs that have identified clusters.

In this study, the solution of three clusters was chosen because they presented different levels of entrepreneurial orientation that helped to perform a good interpretation and to identify their profiles. According to Hair et al. [47] the choice of a cluster solution depends on the evaluation of the researchers and their grounding in the theory of the research area. Table 2 shows the means of the values of the eight indicators of the entrepreneurial orientation scale and the ANOVA test analyses that allow confirming the statistically significant difference between the groups (*p* < 0.001). 

Cluster 1 (group 1) was called “non-entrepreneurs” (*n* = 11; 7.59%) because they presented mean values below the cut-off point of the scale in all indicators, showing a tendency towards disagreement. This group shows a low entrepreneurial orientation in the frequency with which the organization undertakes actions in anticipation of the competition (M = 2.27) and is not usually a pioneer in the introduction of new services or technologies applied to training and/or management, etc. (M = 2.36). Along the same lines, there is a tendency towards disagreement in aspects related to the stimulation of technological, service provision and administrative innovation (M = 2.91) and in creativity and experimentation (M = 2.73). This is a small sample, which may be logical, since COVID-19 has had an impact on the sports sector at all levels and few sports clubs have not undertaken actions or initiatives to address this situation and mitigate the negative effects.

Cluster 2 (group 2) was called “moderate entrepreneurs” representing the largest proportion of the sample of sports clubs interviewed (*n* = 85; 61.38%). This cluster shows different trends in the mean scores of the indicators of the entrepreneurial orientation scale. On the one hand, there are indicators with average values and a positive tendency, such as the fact that the organization gives freedom to individuals and teams to develop new ideas (M = 5.86) or that it emphasizes a policy of delegation to those in charge (M = 5.46). On the other hand, there is a tendency to disagree that the innovative initiatives they promote are difficult for competitors to imitate successfully (M = 3.61).

Cluster 3 (group 3) represents the group of “strong entrepreneurs” (*n* = 49; 31.03%) due to the fact that in all indicators there is a positive tendency towards agreement. They emphasize aspects such as the freedom they give to individuals and teams to develop new ideas (M = 6.37) and the stimulation of creativity and experimentation (M = 6.22). Along the same lines, they are particularly interested in delegating to their managers (M = 6.18) and indicate that their management teams have a strong tendency to be ahead of other organizations in the introduction of new services or ideas (M = 6.16).

### 4.2. Profile of the Groups

After identifying the groups of sports clubs with different levels of entrepreneurial orientation, the profiles of the groups are described below according to other sociodemographic variables of interest to the study. These variables also allow us to ensure the predictive validity of the groups identified in the cluster analysis. Thus, Table 3 describes the percentages and means, according to the type of variable included, of the groups identified.

Within the group of variables related to the characteristics of the sports clubs and the personal characteristics of those responsible, the only variable that contributed to differentiate the groups of sports clubs at a statistically significant level (*p* < 0.05) is the competition level [χ^2^(6) = 10.49, *p* = 0.005]. Non-entrepreneurs are mostly regional-local clubs, while strong entrepreneurs are mostly international-national clubs. Regarding the variables of interest for the study, statistically significant differences were observed between the groups for change in value proposition in the indicator of introduction of new products and services [F(2, 142) = 16.89, *p* = 0.05], change in value delivery [F(2, 142) = 5.90, *p* = 0.003], service quality [F(2, 142) = 11.57, *p* ≤ 0.003], social impact performance [F(2, 142) = 11.57, *p* ≤ 0.001], social cause performance [F(2, 142) = 17. 41, *p* ≤ 0.001], performance in economic impact [F(2, 142) = 17.39, *p* ≤ 0.001] and perceived impact by COVID-19 [F(2, 142) = 6.85, *p* ≤ 0.001].

The non-entrepreneurs (group 1) are characterized, when compared with the other groups, as being mostly local or regional (81.82%), having a higher proportion of clubs with mostly private funding (90.91%), having a number of athletes of less than 100 (45.45%) and being less than 15 years old (45.45%). With respect to the club leaders, there are no significant differences with respect to the other groups, being mostly men (90.91%) with university studies (72.73%), holding the position of directors (63.64%) with a seniority in the club of less than 10 years (54.55%) and in the sports sector of more than 21 years (54.55%).

As for the indicators related to change in value proposition, a negative trend or disagreement was observed in the two indicators: introduction of new products or services (M = 1.36) and reduction in the number of products or services (M =1.82). Post hoc tests showed the existence of statistically significant differences with the other groups, in the case of the first indicator, between this group and the strong entrepreneurs. In the indicators of change in target market (M = 0.82), change in value delivery (M = 1.76) and change in value capture mechanisms (M = 2.10), the same tendency towards disagreement is observed, with significant differences between this group and the strong entrepreneurs, only for the indicator of change in value delivery. On the other hand, this group presents the lowest mean of the three clusters in the indicators of perceived service quality (M = 3.89), performance in social impact (M = 2.79) and performance in social causes (M = 3.22). In the case of service quality, significant differences were only observed when comparing this group with strong entrepreneurs, while in the other two variables, differences were also observed when comparing this group with moderate entrepreneurs. The impact on economic performance variable had the lowest score (M = 4.36) of the three clusters, and the differences between this group and the strong entrepreneurs were statistically significant. Finally, in the case of the impact perceived by the organization as a consequence of the pandemic caused by COVID-19, the same trend is observed (M = 3.09) as in the previous indicators, with significant differences with the strong entrepreneurs.

The moderate entrepreneurs (group 2) are characterized, when compared with the other groups, as being mostly national or international in scope (51.76%), although with a lower proportion than the strong entrepreneurs. Although the proportion is still higher for clubs of a public nature, when compared to the other groups, it has a lower proportion of mostly privately funded clubs (69.41%). The proportion in terms of the number of athletes is similar to that of non-entrepreneurs. On the other hand, there is a higher proportion of clubs older than 16 years (71.09%) than in the case of non-entrepreneurs (54.54%). With respect to the club managers, there are no significant differences with respect to the other groups, the profile being similar to that of the other groups with respect to the proportion of men (84.52%) with university studies (71.43%), holding the position of manager (72.67%) with a seniority in the club of less than 10 years (45.88%), and in the sports sector of more than 21 years (46.99%).

In this cluster, a tendency towards disagreement continues to be observed in the two indicators referring to change in value proposition, although less marked than in the non-entrepreneurs: introduction of new products or services (M = 2.92) and reduction in the number of products or services (M = 2.94). Significant differences were only observed for the first indicator between this group and the strong entrepreneurs. In the indicators of change in target market (M = 1.53), change in value delivery (M = 2.62) and change in value capture mechanisms (M = 2.82), although the trend is less pronounced than in the non-entrepreneurs, a negative trend is still observed, with significant differences between this group and the strong entrepreneurs for the indicator of change in value delivery. On the other hand, in this group there is a change in the trend in the evaluation of service quality (M = 4.65), performance in social causes (M = 4.21) and performance in social impact (M = 3.96), with scores that show a neutral or slightly inclined tendency towards agreement among the interviewees. In all three indicators, significant differences were observed when comparing this group with the strong entrepreneurs. The economic performance impact indicator (M = 5.49) shows a score close to that of the strong entrepreneurs and significantly higher than that of the non-entrepreneurs.

Strong entrepreneurs (group 3) is characterized, when compared to the other groups, by being the group with the highest proportion of national or international clubs (69.39%) and a higher proportion of clubs with mostly private financing (75%), although lower than moderate entrepreneurs. It is the group with the highest proportion of clubs with more than 100 athletes (71.43%) and with a higher proportion of clubs older than 16 years (75%). The profile of the club leaders is mostly men (83.33%) with university studies (74.47%), managers (79.17%) with a seniority in the club of less than 10 years (53.06%), and in the sports sector for more than 21 years (51.02%).

On the other hand, in the change in value proposition variables, they present the highest mean scores, with a tendency towards agreement in the case of the introduction of new products or services (M = 4.88), significantly distanced from the evaluations of the other two groups. For the indicator of reduction in the number of products or services (M = 2.69), a tendency towards disagreement is observed, similar to that of the moderate entrepreneurs. In the indicators of change in target market (M = 2.06), change in value delivery (M = 3.40) and change in value capture mechanisms (M = 3.22), although there is a tendency towards disagreement, it is not as pronounced as in the other clusters. Perceived service quality (M = 5.44), impact on performance in social causes (M = 4.93), performance in social impact (M = 5.13) and economic performance (M = 5.49), show the highest scores of the three clusters significantly. Finally, the impact perceived by the organization as a consequence of the pandemic caused by COVID-19 also has the highest mean score (M = 5.22).

### 4.3. Discriminant Analysis

In order to validate the cluster solution chosen to identify groups of sports clubs with a different entrepreneurial orientation, a discriminant analysis was performed, introducing the proposed cluster solution as the dependent variable and the items of the entrepreneurial orientation scale as independent variables.

Table 4 shows the results derived from the two canonical functions detected by the analysis that explained most of the variance of the dependent variable. The Wilk’s lambda values and the chi-square tests show that the groups of the dependent variable (cluster solution of three groups) differ at a statistically significant level (*p* < 0.001). The canonical correlations between the groups are high and significant (*p* < 0.001), indicating a significant relationship between the functions and belonging to one or the other cluster: 0.90 for function 1 and 0.41 for function 2. The eigenvalue can be interpreted as the proportion of the variance explained by the correlation between the respective canonical variables. Thus, the eigenvalue for function 1 is 4.22, which explains 95.5% of the variance, while the eigenvalue for function 2 is 0.20, which explains 4.5% of the variance.

The classification matrix shows that 97.2% of the cases were correctly classified, with the highest percentage of sports clubs correctly classified for moderate entrepreneurs (96.5%), followed by the strong entrepreneurs’ cluster (91.8%) and non-entrepreneurs (81.8%). The classification matrix provided by the cross-validation was 93.8% of the cases correctly classified. The results confirm the external validity of the proposed three-cluster solution.

## 5. Discussion

This paper analyzes the existence of different profiles of sports clubs according to their level of entrepreneurship in a context of crisis. The clustering technique has been used in several research fields to report on different internally coherent groups [52]. However, although sports entrepreneurs have been the focus of attention of academics and practitioners in recent years [53,54], there is no study that groups them according to their entrepreneurial orientation. This article also provides interesting information by considering the perception of the environment, the adaptation of the business model and the final performance of the organization.

To differentiate these groups, the variable entrepreneurial orientation has been considered, since it is the most widely used concept in the entrepreneurial field [17,55]. This variable has not been used by the organizational entrepreneurship literature to carry out cluster analysis previously, but rather causal variables such as motivations to create or join the organization [56] or outcome variables, such as performance [57]. In this study, three clusters with different organizational, attitudinal and behavioral characteristics are identified: “non-entrepreneurs”, “moderate entrepreneurs”, and “strong entrepreneurs”. Based on other studies, in the absence of a previous conceptualization of clusters, the name of the clusters was proposed based on the authors’ knowledge [52,56].

Each of these clusters presents a different level of entrepreneurial orientation, a variable that aims to create value in the organizations [58]. At the organizational level, knowing how its management and leaders face a situation of uncertainty is a feature that distinguishes an organization and its subsequent performance. However, among the three clusters there are also significant differences in other important variables that can define the organization and its managers.

The three groups differ from each other according to the perceived impact of COVID-19 on the organization. As the academic literature shows, a crisis can have different consequences depending on the identification and management of its effects [59]. The crisis derived from COVID-19 has had important effects on sports organizations [5,11], however, these may be perceived differently according to the organization or the experience of those responsible for it. According to the results obtained in the present study, strong entrepreneurs perceive a considerably greater impact of COVID-19 on their organization than moderate or conservative entrepreneurs. This is understandable, since the sports clubs most affected by COVID-19 may have been forced to reinvent themselves and innovate in order to adapt to the situation [11].

These data may fit with the prospect theory, which proposes that, when faced with the perception of threats in the context, organizations implement a greater number of risky initiatives and entrepreneurial actions [60]. Faced with a context that has had a significant effect on the organization, managers can carry out risky initiatives because there are many possibilities of improving the situation [61]. On the contrary, according to this study, sports organizations that do not perceive a high impact may have decided to maintain their actions and strategies so as not to alter their status quo to a large extent. As can be seen, decision making and subsequent action may depend to a large extent on the individual perception of the environment. Hence, analyzing the perceived impact and subsequent performance of the organization from an entrepreneurial point of view is an important area of study in the academic and professional literature [62].

According to the results obtained, the business model adaptation is a variable that differentiates the three groups of sports clubs. As previous literature shows, after COVID-19, the way in which organizations operate has changed. In this context, adapting the business model can be an opportunity to face a crisis or hostile situation [63]. In the sports context, the limitations and restrictions derived from COVID-19 have forced not-for-profit sports clubs to change their proposal, delivery and value capture to a great extent. According to the results obtained, strong entrepreneurs show significantly higher values in business model adaptation compared to moderate entrepreneurs and non-entrepreneurs. Faced with the situation resulting from the virus, the introduction of new products or services may be one of the most successful actions or initiatives, since it has not been possible to continue with the face-to-face, collective and social value proposition that has been known until now. Strong entrepreneurs introduce new products or services to a greater extent than moderate entrepreneurs and non-entrepreneurs.

Sports clubs, like any other organization, have among their objectives to satisfy the needs of their members, users and athletes. However, needs and expectations may have changed during this hostile and changing situation. It is, therefore, first necessary to identify these needs or expectations and, from there, to adapt their products and services. According to previous studies, non-profit organizations may be more resistant to change compared to for-profit organizations [64]. However, in the current crisis, even non-profit organizations have been forced to take actions to ensure their sustainability and survival. In this context, digitalization and the use of new technologies to reach their target audience have played a fundamental role in the adaptation of the business model [5]. Specifically, the change in the value proposition through the introduction of new products and services has been the adaptation that has had the greatest differentiation among the proposed clusters. This change can be aimed at satisfying users or maintaining their perceived quality.

Similarly, strong entrepreneurs show significantly higher results in value delivery change than non-entrepreneurs and moderate entrepreneurs. Hence, sports clubs with a higher level of entrepreneurship have established closer ties with partners, expanded their relationship to new suppliers, and/or reorganized the sports club. Different studies have analyzed the type of entrepreneurial initiatives, changes or innovations that have been developed in sports clubs [65,66]. However, in this context of social constraints, sports organizations have a limited range of action, so their initiatives must be adjusted to the context. Obviously, the nature of the crisis will significantly condition the type of response possible, just as the nature of the organization will guide the type of response.

According to the results obtained in this study, the tendency is that, in strong entrepreneurs, the level of adaptation of the target market and changes in the mechanisms for capturing value are higher. In this sense, changes in value capture mechanisms are related to the alteration of the price of products or services. This is in line with what was proposed by Musteen et al. [67], who advocated that the entrepreneurial attitude of the organization’s managers is fundamental to successfully plan and implement change. However, despite this trend in the data, change towards a new target market or value capture mechanisms do not present statistically significant differences between the different groups of sport entrepreneurs.

Finally, according to the results obtained, there are significant differences in the three sports club profiles when considering the performance variables analyzed (economic performance, social impact performance, social causes performance, and perceived service quality). Previous literature has largely studied the close relationship between entrepreneurial orientation and the performance of organizations [68], considering it “moderately large” [45] (p. 761). Similarly, although more sparsely, this has been the subject of study in the field of sports. It has been concluded that the performance of sports clubs is closely related to their entrepreneurial orientation [15,44,45]. However, as far as is known, there are no studies that propose different groups of sports entrepreneurs according to entrepreneurship and that their profile considers performance. Hence, the present study has a considerable contribution to the organizational and sports literature.

According to the results obtained, the performance of the three groups of sports clubs is significantly different. The service quality of strong entrepreneurs is significantly higher than that of non-entrepreneurs and moderate entrepreneurs. In the same vein, economic performance also differs greatly between strong entrepreneurs and non-entrepreneurs. This fits with previous literature that exposes that sports clubs with a higher level of entrepreneurial orientation have a higher final performance [11,44]. Innovation and entrepreneurship can transform sports organizations into more competitive entities [69]. Hence, sports clubs with a higher entrepreneurial profile have correspondingly higher financial performance. Therefore, in this challenge for sports clubs to face the crisis and maintain their level of value offering, entrepreneurship is a fundamental factor. “Innovation and risk-taking are dimensions that must be developed because of their wide influence on the final performance of the organization and, consequently, on its economic sustainability” [70] (p. 11).

However, if only the economic or financial end were to be analyzed, a fundamental aspect of sports clubs would remain unconsidered: their social purpose. The marked social function of sports clubs is undeniable, and they contribute significant value to society [71]. This contribution is, if possible, even more important in crisis situations. The social performance of these organizations can be analyzed from two perspectives: (i) performance understood as social impact, i.e., the scope and notoriety of the organization in its environment, and (ii) performance in social causes, understood as its contribution through specific programs to the solution of social problems such as attention to disadvantaged groups or integration through sports [25]. According to the results obtained, strong entrepreneurs have a higher performance in social impact and performance in social causes than non-entrepreneurs and moderate entrepreneurs. Similarly, moderate entrepreneurs have both social performances considerably higher than non-entrepreneurs. From this it can be deduced that the more entrepreneurial Spanish sports clubs have a higher social impact. This fits with what has been previously studied in the associative sports literature [26].

The results reinforce the position that fostering entrepreneurship in organizations not only provides them with a competitive advantage, but also improves their resilience in times of crisis. Let us not forget either that crises are, after all, cyclical phenomena that periodically push social systems to their limits. 

### 5.1. Theorical and Practical Implications

The present study may have interesting theoretical and practical implications due to the fact that there is no similar study in the field of sports. First, a theoretical implication may be the proposal of three groups of entrepreneurs (non-entrepreneurs, moderate entrepreneurs, and strong entrepreneurs) and the description of their profiles. This can provide information on the different profiles in terms of levels of entrepreneurship and business model adaptation, as well as their impact on the final performance. In this turbulent and dynamic context derived from the crisis, academics can use this study as a starting point to continue with the necessary research in the sports sector. Another theoretical contribution can be the consideration of the perceived impact of COVID-19 on the organization. It is often considered that the effect of a hostile situation on the organizations in a sector is equal, but this is not necessarily the case. Even if the impact was identical in all organizations, the perception and identification of the impact would probably be different. Therefore, considering this perception on the part of the organization’s management and decision-makers is a fundamental aspect in decision-making and subsequent action. At the same time, all this can have an important influence on the ability to adapt to the situation and its final performance.

On the other hand, practical implications can be extracted from this study that provide added value. As a first consideration, the diagnosis of an organization’s group, based on its EO, allows us to precisely define features such as its capacity to adapt its business model, its capacity for resilience in crisis situations and to forecast its performance. This can be particularly useful when considering strategies for improving the organization’s performance, especially in the case of non-entrepreneurs and moderate entrepreneurs. It can highlight the need to develop specific actions to increase the entrepreneurial character of the organization through training or the definition of suitable managerial and technical profiles with entrepreneurial traits for the selection of its personnel.

Secondly, the characterization can be useful when assessing the return on any investment made in the organization: private sponsors and governments that finance projects through public aid can anticipate the return that their investment may have depending on the group to which the organization belongs. Improving entrepreneurial capacity may also be a requirement for accessing public resources, together with other features that are becoming increasingly common, such as the professionalization of management and technical staff, etc.

To help sports clubs become progressively more entrepreneurial, structured and targeted training could be carried out in parallel for management and employees. This training would deal with proposals for identifying the internal, external (competition) and market (users and potential users) situation, digitalization and innovation that can have a major impact on the organization, and risk-benefit balance before making important decisions. This could be carried out at the national level by national federations or regional federations.

### 5.2. Limitations and Future Lines of Research

One limitation of the study is the small sample analyzed, which includes only Spanish non-profit sports clubs. This type of sports organization has specific characteristics with respect to their nature. However, in future research it would be interesting to extend the sample to sports organizations of different types and countries. To this end, public and private sports organizations could be analyzed and compared. On the other hand, the variables have been considered through subjective perception instruments and the methodology used is quantitative. As a complement, objective scales could be added as instruments for future studies. In addition, the study could be expanded with qualitative methodologies that could detail the initiatives or actions carried out by the managers or directors. Finally, a limitation that may have arisen due to the high impact that COVID-19 has had—and continues to have—in the sports sector [11], is the low representation of conservative entrepreneurs. Never before has such a high impact been contemplated in the sports sector, perhaps that is why it is more difficult to find sports clubs that perceive a low impact of COVID-19 in their organization and that have practically not undertaken initiatives to address this situation.

## 6. Conclusions

Three dimensions of entrepreneurial orientation were considered to identify the different groups of non-profit sports clubs: innovation, risk-taking, and proactivity. In response to the study objective, three different groups of entrepreneurs in non-profit sports clubs were identified: non-entrepreneurs, moderate entrepreneurs, and strong entrepreneurs. The variables related to the sports club such as size, age of the club and type of financing, and the sociodemographic variables gender, level of education, responsibility in the sports club, length of time in the sports club, and length of time in the sports sector did not contribute to differentiate the groups since there were no significant differences. However, there were significant differences in the competition level. Strong entrepreneurs were characterized by the fact that they were mostly national-international level sports clubs, while non-entrepreneurs were, almost entirely, regional-local level sports clubs.

Similarly, the adaptation of the business model, the perceived impact of COVID-19 on the organization and the economic and social performance and service quality did differ according to the group. Strong entrepreneurs perceive a greater impact of COVID-19 on their organization than non-entrepreneurs and moderate entrepreneurs. Thus, the perception of each manager or management team can have a subsequent impact on decision-making, innovations, risk-taking and proactivity, and, consequently, on their final economic and social performance. Strong entrepreneurs adapt their business model to a greater extent than moderate entrepreneurs and non-entrepreneurs. These differences occur mainly in the change of the value proposition, specifically in the introduction of new products or services. Strong entrepreneurs have introduced products and services to a greater extent than non-entrepreneurs. Similarly, there are differences in the change of value delivery between the different groups. Strong entrepreneurs have strengthened ties with partners, reorganized the sports club and established relationships with new suppliers to a greater extent than non-entrepreneurs and moderate entrepreneurs.

Finally, the performance variables analyzed are also different depending on the sports club group analyzed. Perceived service quality, economic performance, and social performance are significantly higher for strong entrepreneurs than for moderate entrepreneurs and non-entrepreneurs.

## Figures and Tables

**Table 1:** Participants’ description.

Characteristics of Sport Clubs		N	%
Competition level	International-National	80	55.17%
	Regional-Local	65	44.83%
Type of funding	Mostly public	40	27.59%
	Mostly private	105	72.41%
Size (number of athletes)	≤100	54	37.24%
	101–250	53	36.55%
	≥251	38	26.21%
Club age	≤15	42	28.97%
	16–30	41	28.28%
	≥31	62	42.75%
Management team characteristics			
Gender	Male	122	83.14%
	Female	23	15.86%
Educational level	Intermediate studies	42	28.97%
	University studies	103	71.03%
Responsibility/Position	Manager	108	74.49%
	Responsible	37	25.51%
Seniority in the sports club	≤10 years	71	49,0%
	11–20 years	42	29,0%
	≥21 years	32	22,1%
Seniority in sports sector	≤10 years	32	22.07%
	11–20 years	43	29.66%
	≥21 years	70	48.28%

**Table 2:** Average scores for each variable in the three clusters (obtained through the k-averages method).

Items	Cluster 1 (*n* = 11)	Cluster 2 (*n* = 85)	Cluster 3 (*n* = 49)	F	*p* Value
Our organization stresses the use of a fully delegated policy for employees	3.18	5.46	6.18	31.29	<0.001 *
Our organization gives individuals or teams the freedom to develop new ideas	3.36	5.86	6.37	35.94	<0.001 *
In general, the management team of our organization has a strong tendency to be ahead of other organizations in introducing novel services or ideas	3.27	4.79	6.16	51.15	<0.001 *
Our organization encourages and stimulates technological, service delivery and administrative innovation	2.91	4.81	6.04	41.20	<0.001 *
Our organization stimulates creativity and experimentation	2.73	4.84	6.22	55.95	<0.001 *
Our organization’s innovative initiatives are hard for our rivals to successfully imitate	3.18	3.61	5.22	34.09	<0.001 *
In dealing with our rivals, our organization typically initiates actions which they respond to	2.27	3.99	5.59	49.22	<0.001 *
In dealing with our rivals, our organization is very often the first to introduce new services and technologies applied to training and/or management, etc.	2.36	3.88	5.92	73.38	<0.001 *

* Statistically significant mean differences *p* < 0.001.

**Table 3:** Characteristics of the different groups (clusters).

Variable	Response Option	Cluster 1 (*n* = 11)	Cluster 2 (*n* = 85)	Cluster 3 (*n* = 49)
Club characteristics				
Competition level ** χ^2^(2) = 10.49, *p* = 0.005 C^2^ = 0.26	International-National	18.18%	51.76%	69.39% ^(1)^
	Regional-Local	81.82% ^(3)^	48.24%	30.61%
Type of funding χ^2^(2) = 2.44, *p* = 0.30	Mostly public	9.09%	30.59%	25.00%
	Mostly private	90.91%	69.41%	75.00%
Size (nº athletes) χ^2^(4) = 6.94, *p* = 0.14	≤100	45.45%	41.67%	28.57%
	101–250	36.36%	39.29%	32.65%
	≥251	18.18%	19.05%	38.78%
Club age χ^2^(4) = 2.08, *p* = 0.72	≤15	45.45%	28.92%	25.00%
	16–30	18.18%	28.92%	27.08%
	≥31	36.36%	42.17%	47.92%
Characteristics of the management team				
Gender χ^2^(2) = 0.14, *p* = 0.82	Male	90.91%	84.52%	83.33%
	Female	9.09%	15.48%	16.67%
Level of studies χ^2^(2) = 1.37, *p* = 0.93	Intermediate studies	27.27%	28.57%	25.53%
	University studies	72.73%	71.43%	74.47%
Responsibility/Position χ^2^(2) = 1.07, *p* = 0.51	Manager	63.64%	72.62%	79.17%
	Responsible	36.36%	27.38%	20.83%
Seniority in sports club χ^2^(4) = 1.07, *p* = 0.90	≤10 years	54.55%	45.88%	53.06%
	11–20 years	27.27%	29.41%	28.57%
	≥21 years	18.18%	24.71%	18.37%
Seniority in sports sectorχ^2^(4) = 7.21, *p* = 0.12	≤10 years	45.45%	21.69%	16.33%
	11–20 years	0.00%	31.33%	32.65%
	≥21 years	54.55%	46.99%	51.02%
Other variables of interest				
Change in value proposition ***	Introduction of new products or services *** F(2, 142) = 16.89, *p* ≤ 0.001	1.36 (SD ^1^ = 1.86)	2.92 (SD ^1^ = 2.33)	4.88 (SD ^1^ = 2.36)
	Reduction in the number of products or services F(2, 142) = 1.12, *p* = 0.33	1.82 (SD ^1^ = 2.48)	2.94 (SD ^1^ = 2.44)	2.69 (SD ^1^ = 2.24)
Change in target market F(2, 142) = 1.99, *p* = 0.14		0.82 (SD ^1^ = 1.33)	1.53 (SD ^1^ = 1.95)	2.06 (SD ^1^ = 2.39)
Change in value delivery ** F(2, 142) = 5.90, *p* = 0.003		1.76 (SD ^1^ = 1.25)	2.62 (SD ^1^ = 1.68)	3.40 (SD ^1^ = 1.69)
Change in value capture mechanisms F(2, 142) = 0.89, *p* = 0.41		2.10 (SD ^1^ = 2.28)	2.82 (SD ^1^ = 2.62)	3.22 (SD ^1^ = 2.66)
Service quality *** F(2, 142) = 11.57, *p* ≤ 0.001		3.89 (SD ^1^ = 1.22)	4.65 (SD ^1^ = 1.25)	5.44 (SD ^1^ = 0.96)
Social impact performance *** F(2, 142) = 17.41, *p* ≤ 0.001		2.79 (SD ^1^ = 0.95)	3.96 (SD ^1^ = 1.29)	4.93 (SD ^1^ = 1.17)
Social causes performance *** F(2, 142) = 17.39, *p* ≤ 0.001		3.22 (SD ^1^ = 0.85)	4.21 (SD ^1^ = 1.18)	5.13 (SD ^1^ = 1.10)
Economic performance ** F(2, 142) = 6.52, *p* = 0.002		4.36 (SD ^1^ = 1.15)	5.49 (SD ^1^ = 1.31)	5.87 (SD ^1^ = 1.35)
Perceived impact of COVID-19 *** F(2, 142) = 6.85, *p* = 0.001		3.09 (SD ^1^ = 2.12)	4.45 (SD ^1^ = 2.01)	5.22 (SD ^1^ = 1.40)

^1^ SD = Standard deviation; ^2^ C = Contingency coefficient; Indicates statistically significant relationship or statistically significant mean differences ** *p* ≤ 0.01; *** *p* ≤ 0.001; ^(1) (3)^; Results are based on bilateral tests with a level of significance 0.05. For the categorical variables, the results in the table show for each pair of categories the group of clubs with the smallest proportion under the column with the highest proportion.

**Table 4:** Eigenvalues and Wilk’s lambda result for discriminant analysis.

Function	Eigenvalue	% of Variance	Cumulative %	Canonical Correlation	Wilks’ Lambda	Chi-square	*p* Value
1	4.22	95.5	95.5	0.90	0.160	253.90	<0.001 ***
2	0.20	4.5	100.0	0.41	0.834	25.12	0.001 *

Statistically significant mean differences * *p* < 0.05; *** *p* ≤ 0.001

## Data Availability

Not applicable.

## References

[1] Dacombe R. (2013). Sports clubs and civic inclusion: Rethinking the poverty of association. Sport Soc..

[2] Geidne S., Quennerstedt M., Eriksson C. (2013). The youth sports club as a health-promoting setting: An integrative review of research. Scand. J. Public Health.

[3] Kokko S., Selänne H., Alanko L., Heinonen O.J., Korpelainen R., Savonen K., Vasankari T., Kannas L., Kujala U.M., Aira T. (2015). Health promotion activities of sports clubs and coaches, and health and health behaviours in youth participating in sports clubs: The Health Promoting Sports Club study. BMJ Open Sport Exerc. Med..

[4] Johnson S., Vuillemin A., Geidne S., Kokko S., Epstein J., Van Hoye A. (2019). Measuring Health Promotion in Sports Club Settings: A Modified Delphi Study. Health Educ. Behav..

[5] Parnell D., Widdop P., Bond A., Wilson R. (2020). COVID-19, networks and sport. Manag. Sport Leis..

[6] Yeo T.J. (2020). Sport and exercise during and beyond the COVID-19 pandemic. Eur. J. Prev. Cardiol..

[7] Hammami A., Harrabi B., Mohr M., Krustrup P. (2020). Physical activity and coronavirus disease 2019 (COVID-19): Specific recommendations for home-based physical training. Manag. Sport Leis..

[8] Constandt B., Thibaut E., De Bosscher V., Scheerder J., Ricour M., Willem A. (2020). Exercising in Times of Lockdown: An Analysis of the Impact of COVID-19 on Levels and Patterns of Exercise among Adults in Belgium. Int. J. Environ. Res. Public Health.

[9] Francis J., Francis L. (2020). Immunization and participation in amateur youth sports. J. Philos. Sport.

[10] Grix J., Brannagan P.M., Grimes H., Neville R. (2021). The impact of Covid-19 on sport. Int. J. Sport Policy Politics.

[11] Escamilla-Fajardo P., Núñez-Pomar J., Calabuig-Moreno F., Gómez-Tafalla A. (2020). Effects of the COVID-19 Pandemic on Sports Entrepreneurship. Sustainability.

[12] MECS Anuario de Estadísticas Deportivas 2020. http://www.culturaydeporte.gob.es/dam.

[13] (2021). INE. https://www.ine.es/dyngs/INEbase/es/operacion.htm?c=Estadistica_C&cid=1254736176951&menu=ultiDatos&idp=1254735572981.

[14] Ferrando M.G., Goig R.L. (2017). La Popularización del Deporte en España: Encuestas de Hábitos Deportivos 1980–2015.

[15] Núñez-Pomar J.M., Escamilla-Fajardo P., Prado-Gascó V. (2020). Relationship between entrepreneurial orientation and social performance in Spanish sports clubs. The effect of the type of funding and the level of competition. Int. Entrep. Manag. J..

[16] Doern R., Williams N., Vorley T. (2018). Special issue on entrepreneurship and crises: Business as usual? An introduction and review of the literature. Entrep. Reg. Dev..

[17] Wales W.J., Covin J.G., Monsen E. (2020). Entrepreneurial orientation: The necessity of a multilevel conceptualization. Strat. Entrep. J..

[18] Miller D. (1983). The Correlates of Entrepreneurship in Three Types of Firms. Manag. Sci..

[19] Covin J.G., Slevin D.P. (1989). Strategic management of small firms in hostile and benign environments. Strat. Manag. J..

[20] Boudreaux C.J. (2017). Institutional quality and innovation: Some cross-country evidence. J. Entrep. Public Policy.

[21] Bjärsholm D. (2019). Networking as a cornerstone within the practice of social entrepreneurship in sport. Eur. Sport Manag. Q..

[22] Bigliardi B. (2013). The effect of innovation on financial performance: A research study involving SMEs. Innov. Manag. Policy Pract..

[23] Alegre J. (2009). Assessing the impact of organizational learning capability on product innovation performance: An empirical test. Dev. Learn. Organ. Int. J..

[24] Matricano D., Candelo E., Sorrentino M., Martínez-Martínez A. (2019). Absorbing in-bound knowledge within open innovation processes. The case of Fiat Chrysler Automobiles. J. Knowl. Manag..

[25] Martínez-Martínez A., Suárez L.M.C., Montero R.S., Del Arco E.A. (2018). Knowledge management as a tool for improving business processes: An action research approach. J. Ind. Eng. Manag..

[26] Escamilla-Fajardo P., Núñez-Pomar J.M., Gómez-Tafalla A.M. (2020). Exploring Environmental and Entrepreneurial Antecedents of Social Performance in Spanish Sports Clubs: A Symmetric and Asymmetric Approach. Sustainability.

[27] Soininen J., Puumalainen K., Sjögrén H., Syrjä P. (2012). The impact of global economic crisis on SMEs. Manag. Res. Rev..

[28] Laitinen E.K. (2000). Long-term Success of Adaptation Strategies: Evidence from Finnish Companies. Long Range Plan..

[29] Castro P.M., Zermeño M.G. (2020). Being an entrepreneur post-COVID-19 – resilience in times of crisis: A systematic literature review. J. Entrep. Emerg. Econ..

[30] Winby S., Worley C.G. (2014). Management processes for agility, speed, and innovation. Organ. Dyn..

[31] Escamilla-Fajardo P., Núñez-Pomar J.M., Calabuig F. (2021). Does size matter? Entrepreneurial orientation and performance in Spanish sports clubs. Sport Soc..

[32] Miller D. (1996). Configurations revisited. Strateg. Manag. J..

[33] Shepherd D.A. (2020). COVID 19 and Entrepreneurship: Time to Pivot?. J. Manag. Stud..

[34] Cucculelli M., Peruzzi V. (2018). Post-crisis firm survival, business model changes, and learning: Evidence from the Italian manufacturing industry. Small Bus. Econ..

[35] Ritter T., Pedersen C.L. (2020). Analyzing the impact of the coronavirus crisis on business models. Ind. Mark. Manag..

[36] Markides C. (2006). Disruptive Innovation: In Need of Better Theory. J. Prod. Innov. Manag..

[37] Escamilla-Fajardo P., Alguacil M., García-Pascual F. (2021). Business Model Adaptation in Spanish Sports Clubs According to the Perceived Context: Impact on the Social Cause Performance. Sustainability.

[38] Buliga O., Scheiner C.W., Voigt K.I. (2016). Business model innovation and organizational resilience: Towards an integrated conceptual framework. J. Bus. Econ..

[39] Cucculelli M., Bettinelli C. (2015). Business models, intangibles and firm performance: Evidence on corporate entrepreneurship from Italian manufacturing SMEs. Small Bus. Econ..

[40] Saebi T., Lien L., Foss N.J. (2017). What Drives Business Model Adaptation? The Impact of Opportunities, Threats and Strategic Orientation. Long Range Plan..

[41] Ketchen J.D.J., Craighead C.W. (2020). Research at the Intersection of Entrepreneurship, Supply Chain Management, and Strategic Management: Opportunities Highlighted by COVID-19. J. Manag..

[42] Saebi T., Foss N.J., Linder S. (2018). Social Entrepreneurship Research: Past Achievements and Future Promises. J. Manag..

[43] Madella A., Bayle E., Tome J. (2005). The organisational performance of national swimming federations in Mediterranean countries: A comparative approach. Eur. J. Sport Sci..

[44] Hammerschmidt J., Eggers F., Kraus S., Jones P., Filser M. (2020). Entrepreneurial orientation in sports entrepreneurship: A mixed methods analysis of professional soccer clubs in the German-speaking countries. Int. Entrep. Manag. J..

[45] Escamilla-Fajardo P., Núñez-Pomar J.M., González-Serrano M.H. (2020). Sport entrepreneurship in Spanish sports clubs. J. Sport. Econ. Manag..

[46] Vorhies D.W., Morgan N.A. (2005). Benchmarking Marketing Capabilities for Sustainable Competitive Advantage. J. Mark..

[47] Hair J.F., Black W.C., Babin B.J., Anderson R.E. (2014). Multivariate Data Analysis.

[48] Podsakoff N.P. (2003). Common method biases in behavioral research: A critical review of the literature and recommended remedies. J. Appl. Psychol..

[49] Schwarz A., Rizzuto T.E., Carraher-Wolverton C., Roldán J.L., Barrera-Barrera R. (2017). Examining the Impact and Detection of the "Urban Legend" of Common Method Bias. Database Adv. Inf. Syst..

[50] Harman H.H. (1967). Modern Factor Analysis.

[51] Babin B.J., Griffin M., Hair J.F. (2016). Heresies and sacred cows in scholarly marketing publications. J. Bus. Res..

[52] Gudanowska A.E., Kononiuk A., Dębkowska K. (2020). The Application of Cluster Analysis for the Selection of Key Competences of Future-Oriented Entrepreneurs. Eng. Econ..

[53] Radaelli G., Dell’Era C., Frattini F., Petruzzelli A.M. (2018). Entrepreneurship and Human Capital in Professional Sport: A Longitudinal Analysis of the Italian Soccer League. Entrep. Theory Pr..

[54] Hammerschmidt J., Durst S., Kraus S., Puumalainen K. (2021). Professional football clubs and empirical evidence from the COVID-19 crisis: Time for sport entrepreneurship?. Technol. Forecast. Soc. Chang..

[55] Andrade-Valbuena N.A., Merigo-Lindahl J.M., Olavarrieta S.S. (2019). Bibliometric analysis of entrepreneurial orientation. World J. Entrep. Manag. Sustain. Dev..

[56] Cardon M.S., Shinnar R.S., Eisenman M., Rogoff E.G. (2008). Segmenting the population of entrepreneurs: A cluster analysis study. J. Dev. Entrep..

[57] Reid G.C., Smith J.A. (2000). What Makes a New Business Start-Up Successful?. Small Bus. Econ..

[58] Szabo Z.K., Herman E. (2014). Productive Entrepreneurship in the EU and Its Barriers in Transition Economies: A Cluster Analysis. Acta Polytech. Hung..

[59] Pedersen C.L., Ritter T., Di Benedetto C.A. (2020). Managing through a crisis: Managerial implications for business-to-business firms. Ind. Mark. Manag..

[60] Barberis N.C. (2013). Thirty Years of Prospect Theory in Economics: A Review and Assessment. J. Econ. Perspect..

[61] Chattopadhyay P., Glick W.H., Huber G.P. (2001). Organizational Actions in Response to Threats and Opportunities. Acad. Manag. J..

[62] Devece C., Peris-Ortiz M., Rueda-Armengot C. (2016). Entrepreneurship during economic crisis: Success factors and paths to failure. J. Bus. Res..

[63] Seetharaman P. (2020). Business models shifts: Impact of Covid-19. Int. J. Inf. Manag..

[64] Hull C.E., Lio B.H. (2006). Innovation in non-profit and for-profit organizations: Visionary, strategic, and financial considerations. J. Chang. Manag..

[65] Hoeber L., Hoeber O. (2012). Determinants of an Innovation Process: A Case Study of Technological Innovation in a Community Sport Organization. J. Sport Manag..

[66] Hoeber L., Doherty A., Hoeber O., Wolfe R. (2015). The nature of innovation in community sport organizations. Eur. Sport Manag. Q..

[67] Musteen M., Barker V.L., Baeten V.L. (2010). The Influence of CEO Tenure and Attitude Toward Change on Organizational Approaches to Innovation. J. Appl. Behav. Sci..

[68] Engelen A., Gupta V., Strenger L., Brettel M. (2015). Entrepreneurial Orientation, Firm Performance, and the Moderating Role of Transformational Leadership Behaviors. J. Manag..

[69] Rauch A., Wiklund J., Lumpkin G.T., Frese M. (2009). Entrepreneurial Orientation and Business Performance: An Assessment of Past Research and Suggestions for the Future. Entrep. Theory Pr..

[70] Escamilla-Fajardo P., Alguacil M., Gómez-Tafalla A. (2021). Effects of Entrepreneurial Orientation and Passion for Work on Performance Variables in Sports Clubs. Sustainability.

[71] Waardenburg M. (2016). Which wider social roles? An analysis of social roles ascribed to voluntary sports clubs. Eur. J. Sport Soc..

